# Supplementary material for: Structure, Vibrational Spectra, and Cryogenic MatrixPhotochemistry of 6-Bromopyridine-2-carbaldehyde: From the Single Molecule of the Compound to the Neat Crystalline Material
Source: Molecules. 2023 Feb 9;28(4):1673. doi: 10.3390/molecules28041673 (PMC9962366; doi:10.3390/molecules28041673)
Supplement: Supplementary file 1 [file molecules-28-01673-s001.zip › molecules-2194262-supplementary.pdf]

Supplementary Material

Structure, Vibrational Spectra, and Cryogenic Matrix Photochemistry of 6-Bromopyridine-2-Carbaldehyde: From the Single Molecule of the Compound to a the Neat Crystalline Material

Anna Luiza B. Brito [1,*], Susy Lopes [1], Gulce Ogruc Ildiz [1,2] and Rui Fausto [1,*]

[1] CQC-IMS, Department of Chemistry, University of Coimbra, 3004-535 Coimbra, Portugal
[2] Department of Physics, Faculty of Science and Letters, Istanbul Kultur University, Atakoy Campus, Bakirkoy 34156, Istanbul, Turkey
* Correspondence: anna.brito@qui.uc.pt (A.L.B.B.); rfausto@ci.uc.pt (R.F.)

Index

Figure S1. Different views of the packing for crystalline BPCA
Figure S2. Mass spectrum of BPCA, with peak assignments
Table S1. Definition of the symmetry coordinates used in the BPCA normal coordinate analysis.
Table S2. Definition of the symmetry coordinates used in the 2-bromopyridine normal coordinate analysis.
Table S3. DFT(B3LYP)/6-311++G(d,p) Cartesian coordinates for the optimized structures of the *trans* and *cis* conformers of BPCA

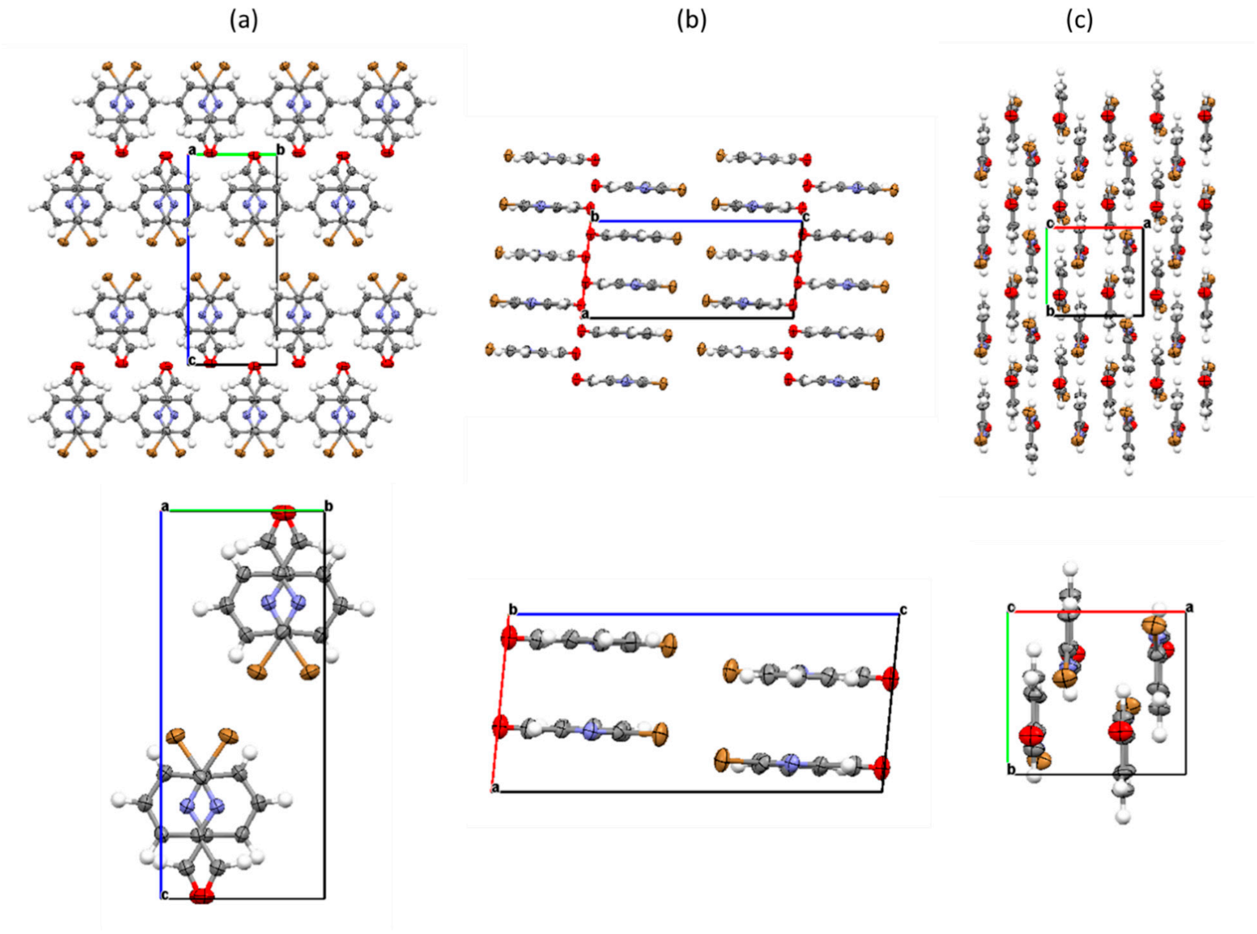

Figure S1. Packing for crystalline BPCA viewed along the crystallographic axes *a* (a), *b* (b) and *c* (c). Top images are expanded regions of the crystal, while the lower images correspond to the unit cell. Color codes: hydrogen: white; carbon: grey; nitrogen: blue; oxygen: red; bromine: orange. Crystallographic axes *a*, *b* and *c* are shown in red, green and blue color, respectively. The figures were drawn using the information in the CIF file CCDC reference: 608389. [14]

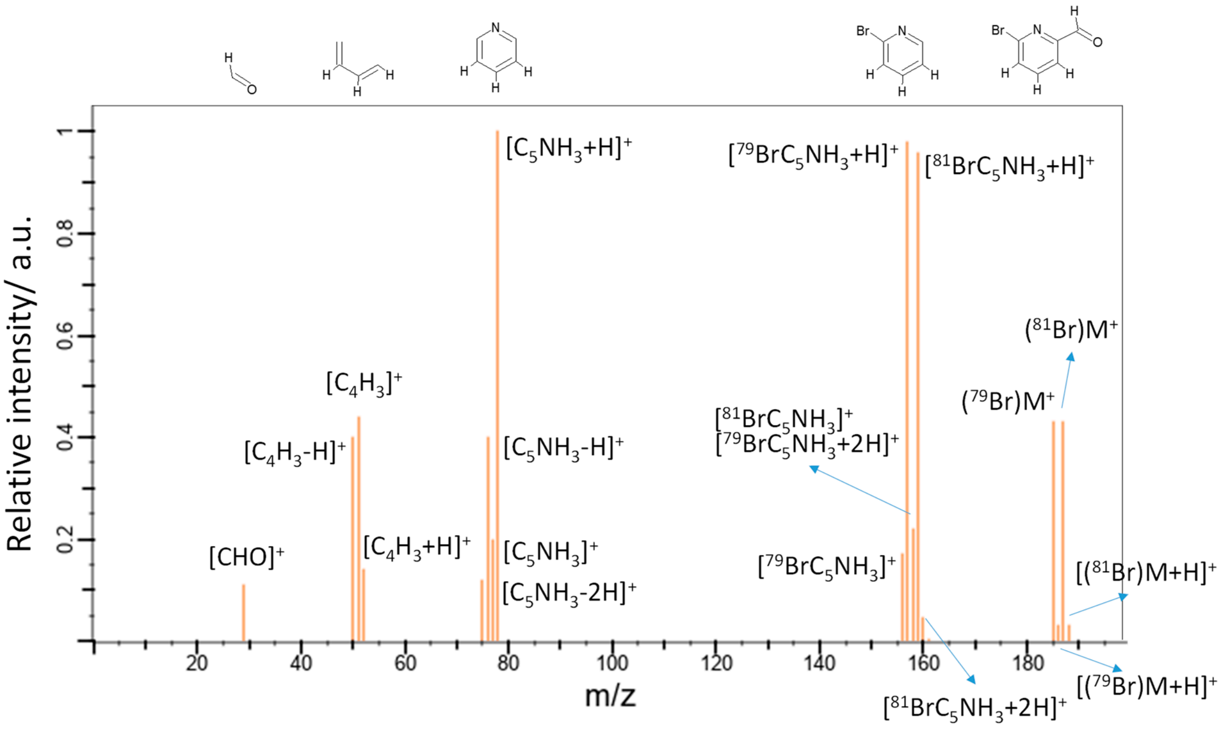

Figure S2. Mass spectrum of BPCA,[15] with peak assignments. Representations of the base-fragments associated with the different groups of peaks are shown at the top of the figure above the corresponding peaks.

Table S1. Definition of the symmetry coordinates used in the normal coordinate analysis performed on the *trans* and *cis* conformers of BPCA.[a]

Coordinate	Symmetry	Approximate Description	Definition[b]
S_1	A′	nC=O	nC=O
S_2	A′	nC–H_{al}	nC–H_{al}
S_3	A′	nC–C	nC–C
S_4	A′	nC–Br	nC–Br
S_5	A′	nC2/3–H s	nC2–H + nC3–H
S_6	A′	nC2/3–H a	nC2–H – nC3–H
S_7	A′	nC4–H	nC4–H
S_8	A′	nring1	–nNC2 + 2nC2C3 – nC3C4 – nC4C5 + 2nC5C6 – nC6N
S_9	A′	nring2	nNC2 – nC3C4 + nC4C5 – nC6N
S_{10}	A′	nring3	nNC2 + nC2C3 + nC3C4 + nC4C5 + nC5C6 + nC6N
S_{11}	A′	nring4	–nNC2 – 2nC2C3 – nC3C4 + nC4C5 + 2nC5C6 + nC6N
S_{12}	A′	nring5	nNC2 – nC3C4 – nC4C5 + nC6N
S_{13}	A′	nring6	nNC2 – nC2C3 + nC3C4 – nC4C5 + nC5C6 – nC6N
S_{14}	A′	dC=O	2dCC=O – dCCH_{al}– dO=CH_{al}
S_{15}	A′	dCH_{al}	dCCH_{al}– dO=CH_{al}
S_{16}	A′	dCBr	dNCBr – dCCBr
S_{17}	A′	dCH_r 1	dC2C3H – dC4C3H + dC3C4H – dC5C4H + dC4C5H – dC6C5H
S_{18}	A′	dCH_r 2	dC2C3H – dC4C3H – 2dC3C4H + 2dC5C4H + dC4C5H – dC6C5H
S_{19}	A′	dCH_r 3	dC2C3H – dC4C3H – dC4C5H + dC6C5H

S_{20}	A′	wCHO	dNC2C11 – dC3C2C11
S_{21}	A′	dring1	dNC3C4 – dC2C3C4 + dC3C4C5 – dC4C5C6 + dC5C6N – dC6NC2
S_{22}	A′	dring2	–dNC2C3 – dC2C3C4 + 2dC3C4C5 – dC4C5C6 – dC5C6N + 2dC6NC2
S_{23}	A′	dring3	dNC2C3 – dC2C3C4 + dC4C5C6 – dC5C6N
S_{24}	A′′	CH_{al}	H_{al}–(CC11O)
S_{25}	A′′	CHO	C11–(NC2C3)
S_{26}	A′′	CBr	Br–(C5C6N)
S_{27}	A′′	CH_r 1	H–(C2C3C4) + H–(C3C4C5) + H–(C4C5C6)
S_{28}	A′′	CH_r 2	H–(C2C3C4) – 2 H–(C3C4C5) + H–(C4C5C6)
S_{29}	A′′	CH_r 3	H–(C2C3C4) – H–(C4C5C6)
S_{30}	A′′	C–CHO	NCC=O + CCC=O + $NCCH_{al}$ + $CCCH_{al}$
S_{31}	A′′	ring1	2 NC2C3C4 + C2C3C4C5 + C3C4C5C6 + 2 C4C5C6N + C5C6NC2 + C6NC2C3
S_{32}	A′′	ring2	NC2C3C4 – C2C3C4C5 + C3C4C5C6 – C4C5C6N + C5C6NC2 – C6NC2C3
S_{33}	A′′	ring3	C2C3C4C5 – C3C4C5C6 + C5C6NC2 – C6NC2C3

[a] Abbreviations: n = stretching, d = in-plane bending, g = out-of-plane rocking, w, wagging; t = torsion; s = symmetric; a = anti-symmetric; al = aldehyde; r, ring. See Figure 1 for the atom numbering scheme. [b] Normalization factors are not included; they are calculated as $\sqrt{S c_i^2}$, where c_i are the coefficients of combination of the coordinates.

Table S2. Definition of the symmetry coordinates used in the normal coordinate analysis performed on 2-bromopyridine.[a.]

Coordinate	Symmetry	Approximate Description	Definition[b]
S_{1}	A′	nC–Br	nC–Br
S_{2}	A′	nCH$_{r}$ 1	nC2–H + nC3–H + nC4–H + nC5–H
S_{3}	A′	nCH$_{r}$ 2	nC2–H + nC3–H – nC4–H – nC5–H
S_{4}	A′	nCH$_{r}$ 3	nC2–H – nC3–H + nC4–H – nC5–H
S_{5}	A′	nCH$_{r}$ 4	nC2–H – nC3–H – nC4–H + nC5–H
S_{6}	A′	nring1	–nNC2 + 2nC2C3 – nC3C4 – nC4C5 + 2nC5C6 – nC6N
S_{7}	A′	nring2	nNC2 – nC3C4 + nC4C5 – nC6N
S_{8}	A′	nring3	nNC2 + nC2C3 + nC3C4 + nC4C5 + nC5C6 + nC6N
S_{9}	A′	nring4	–nNC2 – 2nC2C3 – nC3C4 + nC4C5 + 2nC5C6 + nC6N
S_{10}	A′	nring5	nNC2 – nC3C4 – nC4C5 + nC6N
S_{11}	A′	nring6	nNC2 – nC2C3 + nC3C4 – nC4C5 + nC5C6 – nC6N
S_{12}	A′	dCBr	dNCBr – dCCBr
S_{13}	A′	dCH$_{r}$ 1	dNCH – dC3C2H + dC2C3H – dC4C3H + dC3C4H – dC5C4H + dC4C5H – dC6C5H
S_{14}	A′	dCH$_{r}$ 2	dNCH – dC3C2H + dC2C3H – dC4C3H – dC3C4H + dC5C4H – dC4C5H + dC6C5H
S_{15}	A′	dCH$_{r}$ 3	dNCH – dC3C2H – dC2C3H + dC4C3H + dC3C4H – dC5C4H – dC4C5H + dC6C5H
S_{16}	A′	dCH$_{r}$ 4	dNCH – dC3C2H – dC2C3H + dC4C3H – dC3C4H + dC5C4H + dC4C5H – dC6C5H
S_{17}	A′	dring 1	dNC3C4 – dC2C3C4 + dC3C4C5 – dC4C5C6 + dC5C6N – dC6NC2
S_{18}	A′	dring 2	–dNC2C3 – dC2C3C4 + 2dC3C4C5 – dC4C5C6 – dC5C6N + 2dC6NC2
S_{19}	A′	dring 3	dNC2C3 – dC2C3C4 + dC4C5C6 – dC5C6N
S_{20}	A′′	CBr	Br–(C5C6N)
S_{21}	A′′	CH$_{r}$ 1	H–(NC2C3) + H–(C2C3C4) + H–(C3C4C5) + H–(C4C5C6)
S_{22}	A′′	CH$_{r}$ 2	H–(NC2C3) + H–(C2C3C4) – H–(C3C4C5) – H–(C4C5C6)
S_{23}	A′′	CH$_{r}$ 3	H–(NC2C3) – H–(C2C3C4) + H–(C3C4C5) – H–(C4C5C6)
S_{24}	A′′	CH$_{r}$ 4	H–(NC2C3) – H–(C2C3C4) – H–(C3C4C5) + H–(C4C5C6)
S_{25}	A′′	ring1	2 NC2C3C4 + C2C3C4C5 + C3C4C5C6 + 2 C4C5C6N + C5C6NC2 + C6NC2C3
S_{26}	A′′	ring2	NC2C3C4 – C2C3C4C5 + C3C4C5C6 – C4C5C6N + C5C6NC2 – C6NC2C3
S_{27}	A′′	ring3	C2C3C4C5 – C3C4C5C6 + C5C6NC2 – C6NC2C3

[a] Abbreviations: n = stretching, d = in-plane bending, = out-of-plane rocking, = torsion; r, ring. [b] Normalization factors are not included; they are calculated as $\sqrt{Sc_i^2}$, where c_i are the coefficients of combination of the coordinates.

Table S3. DFT(B3LYP)/6-311++G(d,p) Cartesian coordinates (Å) for the optimized structures of the *trans* and *cis* conformers of BPCA.

	trans			*cis*		
	x	*y*	*z*	*x*	*y*	*z*
N1	–0.365040	–0.693491	0.000000	–0.045027	–0.767440	0.000000
C2	0.605220	–1.624375	0.000000	1.123598	–1.432824	0.000000
C3	1.962531	–1.310469	0.000000	2.357679	–0.787247	0.000000
C4	2.323579	0.034331	0.000000	2.380769	0.608764	0.000000
C5	1.328209	1.005394	0.000000	1.179187	1.299719	0.000000
C6	0.000000	0.567267	0.000000	0.000000	0.539129	0.000000
H7	2.693950	–2.108504	0.000000	3.275832	–1.363314	0.000000
H8	3.366943	0.328154	0.000000	3.320581	1.148601	0.000000
H9	1.562395	2.061098	0.000000	1.141200	2.380376	0.000000
Br10	–1.403905	1.882891	0.000000	–1.683096	1.474383	0.000000
C11	0.129597	–3.040859	0.000000	1.082486	–2.929967	0.000000
H12	–0.972075	–3.140848	0.000000	2.088915	–3.401298	0.000000
O13	0.868241	–3.996798	0.000000	0.081839	–3.597639	0.000000

References:

14. Zhang, H.-X.; Qin, D.-B.; Jing, L.-H.; Gua, S.-J.; Mao, Z.-H. 6-Bromopyridine-2-carbaldehyde. *Acta Cryst. E Struct. Rep.* **2006**, *62*, o1715–o1716.
